# Induced Wide Nematic Phase by Seven-Ring Supramolecular H-Bonded Systems: Experimental and Computational Evaluation

**DOI:** 10.3390/molecules25071694

**Published:** 2020-04-07

**Authors:** Latifah Abdullah Alshabanah, Laila A. Al-Mutabagani, Hoda A. Ahmed, Mohamed Hagar

**Affiliations:** 1 Chemistry Department, College of Science, Princess Nourah bint Abdulrahman University, Riyadh 11671, Saudi Arabia; Laalsabanah@pnu.eud.sa (L.A.A.); Laalmutbagani@pnu.edu.sa (L.A.A.-M.); 2 Department of Chemistry, Faculty of Science, Cairo University, Cairo 12613, Egypt; 3 Chemistry Department, College of Sciences, Yanbu, Taibah University, Yanbu 30799, Saudi Arabia; 4 Chemistry Department, Faculty of Science, Alexandria University, Alexandria 21321, Egypt

**Keywords:** supramolecular H-bonding complexes, dinitrogen H-boning complexes, nematic stability, DFT calculations, photophysical

## Abstract

New seven-ring systems of dipyridine derivative liquid crystalline 2:1 supramolecular H-bonded complexes were formed between 4-n-alkoxyphenylazo benzoic acids and 4-(2-(pyridin-4-yl)diazenyl)phenyl nicotinate. Mesomorphic behaviors of the prepared complexes were investigated using a combination of differential scanning calorimetry (DSC) and polarizing optical microscopy (POM). Fermi bands attributed to the presence of intermolecular H-bond interactions were confirmed by FT–IR spectroscopy. All prepared complexes possessed an enantiotropic nematic phase with a broad temperature nematogenic range. Phases were confirmed by miscibility with a standard nematic (N) compound. A comparison was constructed to investigate the influence of the incorporation of the azophenyl moiety on the mesomeric behavior of corresponding five-membered complexes. It was found that the present complexes observed induced a wide nematic phase with relatively higher temperature ranges than the five aromatic systems. Density functional theory (DFT) suggested the nonlinear geometry of the formed complex. The results of the DFT explained the nematic mesophase formation. Moreover, the π–π stacking of the aromatic moiety in the phenylazo acid plays an effective role in the mesomorphic thermal stability. The energy difference between the frontier molecular orbitals, HOMO (highest occupied) and LUMO (lowest occupied), and the molecular electrostatic potential (MEP) of the prepared complexes were estimated by DFT calculations. The results were used to illustrate the observed nematic phase for all H-bonded supramolecular complexes. Finally, photophysical studies were discussed which were carried out by UV spectroscopy connected to a hot stage.

## 1. Introduction

In the last few years, interesting studies of heterocyclic liquid crystal compounds comprising azopyridene moieties have been prepared and their mesomorphic properties were characterized [1,2,3,4,5,6,7,8,9,10,11]. The incorporation of heterocyclic rings with electronegative hetero-atoms (N, O or S) will enhance and impact powerful polar induction [12]. The conjugation of heterocyclic mesogens offered organic photonics applications powers of heterocyclic mesogens offered organic photonics applications [13]. In fact, the ability of trans-cis-isomerization upon thermal and photoirradiation of azopyridene liquid crystals (LCs) makes them good candidates due to their photo-responsiveness [14,15], making it interesting to study such properties. Recently, azopyridene has been used in the formation of nanofiber supramolecular self-assembling and hydrogen/halogen-bonding LCs with photo-induced transition phenomena [16,17,18,19,20]. Designing of photosensitive supramolecular liquid crystal materials (SMLCs) through intermolecular hydrogen-bonding interactions are also concerns of our area of interest [6,21,22,23,24,25,26]. The use of heterocyclic rings (dipyridine) with different nitrogen atom orientations widely improves many characteristics of SMLCs complexes [27,28]. Changes in the structural and molecular characteristics may influence mesomorphic properties essential for technical use. A combination of rigid (aromatic) and flexible segments (alkyl chains) are essential in designing anisotropic LCs architectures with better phase transitions. Recently, the construction of materials according to previous computational predictions has received high attention from many researchers [1,29,30,31,32,33,34,35,36,37,38]. The mutual influence of many optical parameters requires stimulated information regarding the energies of molecular orbitals as well as the molecular geometries of designed compounds. Very recently, our research group [4,10,22,39,40,41,42,43,44,45,46,47] focused on the computational parameters of new synthesized supramolecular liquid crystal (SMLC) systems by illustrating the behavior of the supramolecular H-bonded complexes of carboxylic acids and pyridine derivatives experimentally and theoretically. The molecular shape and alkoxy chain length (as terminals) play an important role in the formation, thermal stability, type, mesomorphic range, as well as estimated geometrical and thermal parameters of liquid crystalline architectures. The molecules tend to be oriented in a parallel arrangement as the length of the terminal substituent increases [48]. In addition, the terminal chain length plays an important role in influencing the heliconical and twist–bend nematic phases [49,50].

**Scheme 1:**
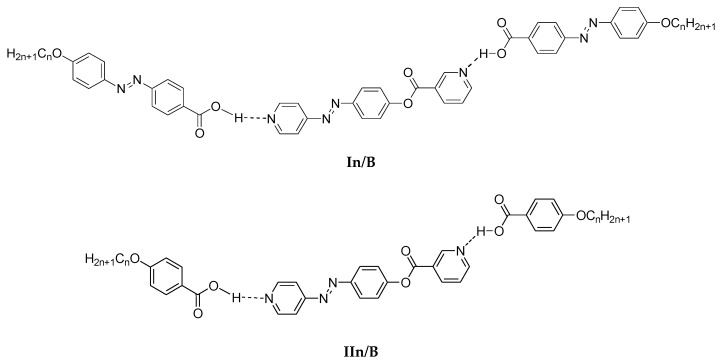
2:1 Supramolecular hydrogen bonded complexes **In/B** and **IIn/B**.

Based on the above context, the aim of the present study is to design new seven-ring architectures of 2:1 supramolecular H-bonded (SMHB) complexes based on dipyridine and phenyl nicotinate as base moieties (B), and 4-n-alkoxyphenylazobenzoic acids as H-donor moieties (In). The purpose of this study is to investigate their mesomorphic and optical properties as well as their geometrical parameters. Moreover, we studied theoretical (DFT) calculations to understand the experimental variables that could be affected by the possible orientation of dinitrogen atoms for the predicted conformers which impacts the degree of the intermolecular interaction between the SMHBCs. Further, a comparison is established between the present seven-membered systems and previous five aromatic ring SMHB complexes (IIn/B, Scheme 1) to investigate the effect of incorporation of an additional phenylazo group on estimated experimental and theoretical parameters.

## 2. Experimental

The dinitrogen base **B** was synthesized according to the procedure mentioned in the Supplementary Data.

### Preparation of 2:1 SMHB Complexes

**Scheme 2:**
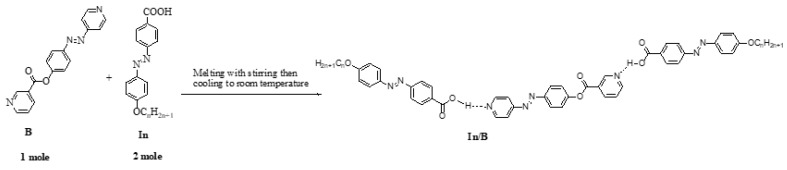
Preparation of 2:1 supramolecular H-bonded complexes (**In/B**).

Supramolecular **In/B** complexes were prepared from 2:1 molar ratios of alkoxyphenylazobezoic acids (**In**) with different chain lengths from n = 6 to n = 12 and a dinitrogen base (**B**), respectively. The solid mixture was melted together in dry watch glass at ≈ 270 °C with stirring to form an intimate blend and then allowed to cool to room temperature (Scheme 2). The formation of supramolecular complexes (**In/B**) was proved by DSC investigation as well as FT–IR spectroscopy.

## 3. Results and Discussion

### 3.1. FT–IR Characterizations of 2:1 SMHB Complexes

The mixing of an acid and a base affords four possible kinds of products: (a) a mixture of non-ionized acids and bases; (b) the H-bonding supramolecular complexes; (c) pure organic ionic salts; and (d) salts in association with H-bonded supramolecular complexes [51]. However, these mixtures could be confirmed with the FT–IR spectroscopy. The pure ionic salts could be confirmed by the presence of the C=O stretching vibration lower than 1700 cm^−1^. On the other hand, the H-bonded formation increases the C=O stretch vibration more than that of the free acid. Finally, the case such mixing could be proven by the presence of two absorption peaks, one higher than 1700 cm^−1^ and the other lower. In the present study, the absence of the lower stretching frequency <1700 cm^–1^ is evidence of the presence of H-bonded supramolecular complexes as the only product [52,53]. 

**Figure 1:**
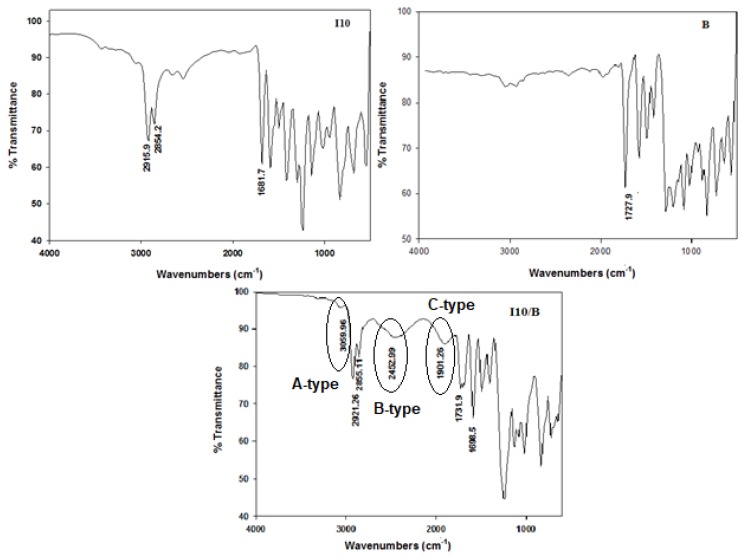
FT–IR spectra of **B, I10** and their supramolecular **I10/B** complex.

The formation of the dinitrogen compound and the azo acid was confirmed by FT–IR spectral data measurements for the individual components as well as to their supramolecular complexes. The FT–IR spectrum of **I10, B** and their complex **I10/B** (as representative examples) are given in Figure 1. The signal at 1681 cm^−1^ was assigned to the dimeric C=O group of the azo acid. The dimeric H-bond between the azo acids is replaced with that between the azo acid (**I10**). The nitrogen of dipyridine (**B**) could be confirmed by the FT–IR measurements. An important piece of evidence concerning the H-bonded supramolecular complexes formation is the C=O, OH stretching vibration. It has been reported [38,54,55,56,57,58,59] that the presence of three Fermi resonance vibration bands of the H-bonded OH groups **A**-, **B**- and **C**-types provides evidence for the supramolecular complex formation. The **A**-type Fermi band of complex **I10/B** is overlapped with that of the C–H vibrational peaks at 2921 to 2855 cm^−1^. Moreover, the peak at 2452 (**I10/B**) and could be assigned to **B**-type of the in-plane bending vibration of the O–H. On the other hand, 1901 cm^−1^ is corresponding to the **C**-type Fermi band due to the interaction between the overtone of the torsional effect and the fundamental stretching vibration of the OH. 

### 3.2. Mesomorphic Study of 2:1 SMHBLC Complexes 

**Figure 2:**
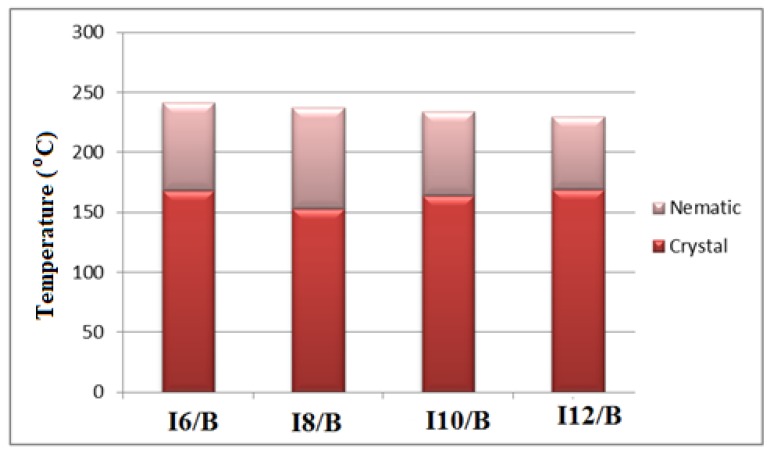
Graphical DSC transitions of the 2:1 supramolecular complex series, **In/B**.

**Figure 3:**
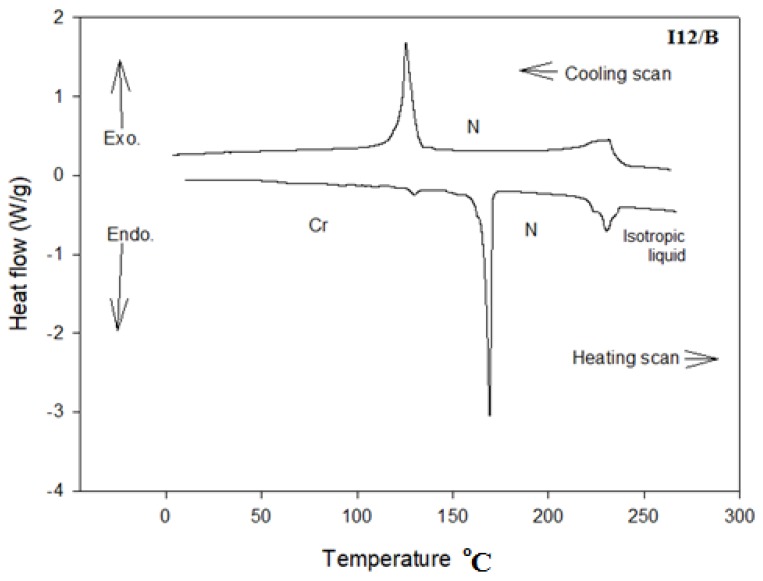
DSC thermograms on the second heating/cooling cycles of the 2:1 supramolecular H-bonded (SMHB) complex **I12/B** at a rate of 10 °C/min.

**Figure 4:**
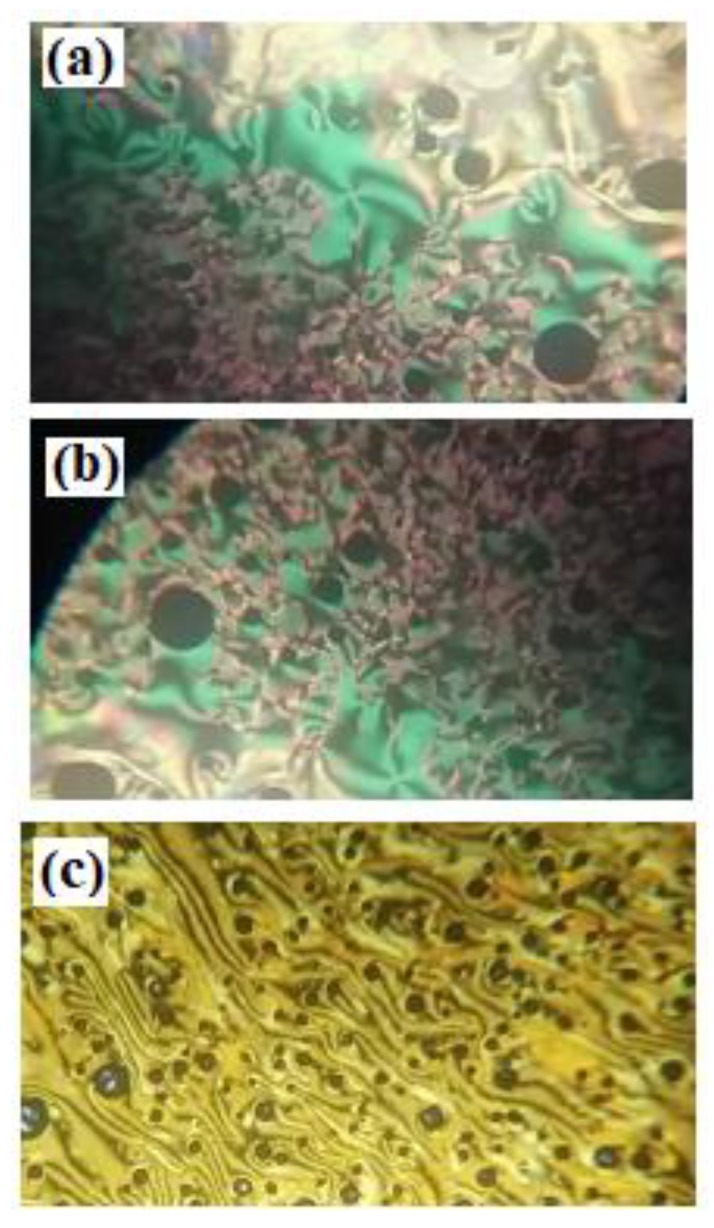
Nematic polarizing optical microscopy (POM) textures on heating for 2:1 supramolecular complexes (**a**) **I12/B** at 218.0 °C, (**b**) **I12/B** at 225.0 °C (**c**) and **I8/B** at 180.0 °C phases.

Interestingly, mesomorphic and optical behaviors of the seven-ring 2:1 supramolecular H-bonded systems (**In/B**) were analyzed. Details of the transition temperatures and their associated enthalpy, as well as the normalized entropy of the mesophase transition for all investigated SMHB **In/B** complexes, as derived from DSC measurements, are summarized in Table 1. Transition temperatures of the investigated complexes are represented graphically in Figure 2 in order to evaluate the effect of terminal alkoxy chain lengths of acid components on the mesophase behavior. During heating and cooling scans, all SMLHB complexes showed only enantiotropic nematic phase. The POM measurements revealed textures which confirmed the thread/schlieren texture of the nematic mesophase (Figure 3). In order to confirm that the N phase is the only mesophase exhibited by all complexes, miscibility studies were constructed between the present complexes and the standard nematic 4-hexyloxy benzoic acid, resulting in only an enantiotropic N phase. Representative DSC thermograms on the second heating/cooling cycles of supramolecular **I12/B** complexes are shown in Figure 4.

The evaluated data of Table 1 and Figure 2 revealed that enantiotropic nematic mesophase was only observed for all investigated 2:1 mixtures and their thermal stabilities declined by increasing the terminal alkoxy chain length of the alkoxyphenylazobezoic acid component from n = 6 to 12. Additionally, the melting point transitions have irregular trends. It should be mentioned that the thermal and optical result of dipyridine derivative **B** is nonmonomorphic and converted directly from solid crystal to isotropic liquid at 148.1 °C. Meanwhile, the pure alkoxyphenylazobezoic acid derivatives **In** exhibit a smectic C phase (SmC) with relatively high transition temperatures and nematic phase (N) with a very narrow range [60]. Despite that the dipyridine compound **B** did not exhibit any phases, all prepared 2:1 supramolecular **In/B** complexes observed an induced wide nematic phase with relatively higher value temperature ranges for the complex **I8/B** (~84.3 °C) and lower values for **I12/B** (~60.9 °C). In addition, the nematic transition stability decreases with the increment of the chain attached to the acid complement (**n**); thus, the length and the core of the H-donor molecule is found to be more dominant on the stability of the observed N phase. It can be concluded that the increment of the molecular anisotropy in the SMHB complexes promotes the broadening of nematic phases that agree with the previous work [22] which revealed that the increase of the mesogenic part length enhances the stability of nematic phases. The nematic range (*Δ*T_N_ = T_N_-T_cr_) is decreased in the order **I8/B** > **I6/B** > **I10/B** > **I12/B**. Supramolecular complex **I8/B** has the highest broad mesomorphic range. The descending trend in the N thermal stability with terminal chain length **n** can be concluded to the dilution of the rigid mesogenic core [61,62]. This could be due to the higher van der Waals attractions between the longer alkoxy chain length leading to the intertwining and facilitates terminal interaction and alkyl group aggregation which are important effects for the nematic phase enhancement.

**Figure 5:**
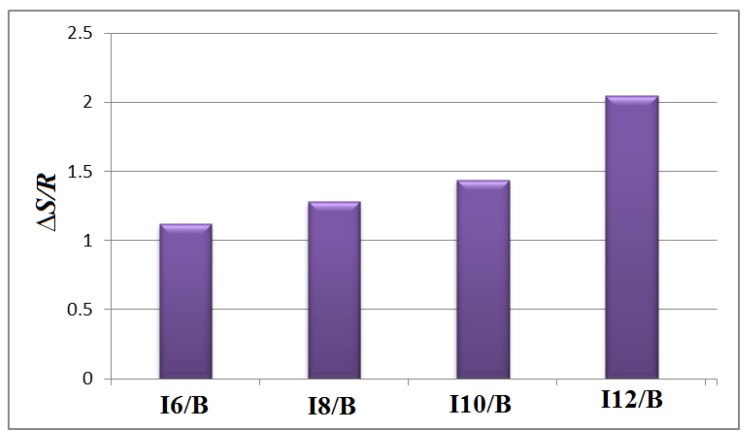
Normalized entropy changes with the alkoxy chain length of SMHB **In/B** complexes.

The normalized entropy, driven form DSC, of transitions (***ΔS/R***) were calculated for the present supramolecular **In/B** complexes and the results are tabulated in Table 1. The results indicate an increment of the entropy change with the terminal alkoxy chain length (Figure 5) that reflects the decrease in the biaxiality of the mesogenic part, resulting in an increase in conformational entropy [55]. Moreover, the variation in the entropy change with alkoxy chain length may be attributed to the change of molecular interactions between molecules, which are affected by the dipole moment, polarizability, rigidity, aspect ratio (length/breadth ratio) and geometrical shape of molecules. These factors may contribute to the conformational, orientational and translational entropies of the molecule in different amounts. The increase of ***ΔS/R*** values when increasing the number of carbons in the alkoxy chain is probably due to the disappearance of the long orientational order and increase of the number of conformational distributions at the mesophase transitions. 

**Figure 6:**
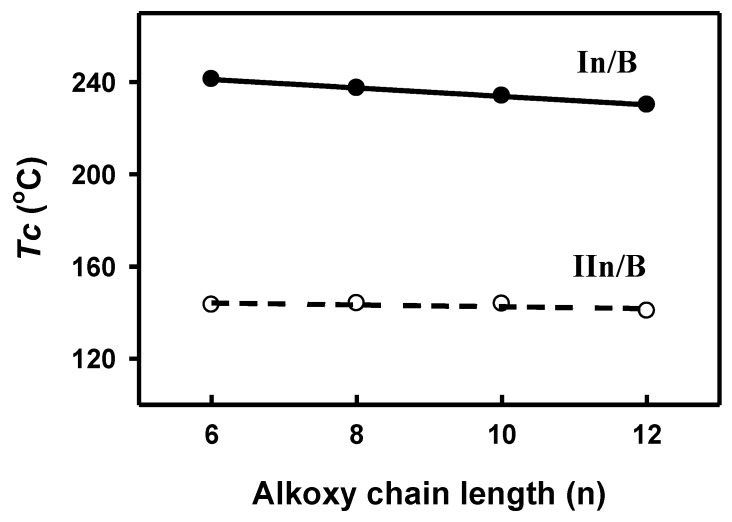
Comparison between the present SMHB **In/B** complexes (●) and five-ring **IIn/B** system (○) in nematic stability.

In order to investigate the effect of the incorporation of an extra phenylazo group part to the acid moiety on the mesomorphic behavior, a comparison was constructed between the mesophase stability (T**_C_**) of the present investigated seven-ring **In/B** complexes with the data reported of the previously prepared five-ring SMHB complexes **IIn/B** [63] (Figure 6). As can be seen from Figure 4, the addition of the extra phenylazo group into **IIn/B** resulted in a significant increase in the Tc nematic values. The nematic stability (T_N_) values were found to increase in the range of 97.9, 93.4, 90.3 and 89.4 °C for alkoxy acid chains n = 6, 8, 10 and 12, respectively. These increments are attributed to the increase of polarizability of the whole seven-aromatic-ring molecules as well as the increase of rigidity and the aspect ratio, which, in turn, lead to an increase of intermolecular interactions between molecules.

### 3.3. DFT Calculations 

#### 3.3.1. Molecular Geometries

**Figure 7:**
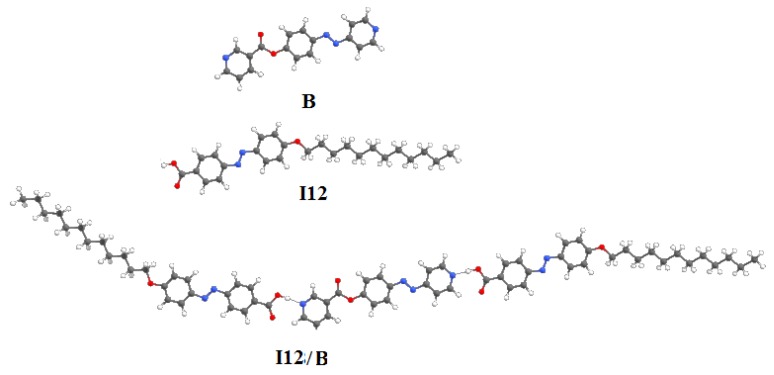
The calculated geometry of **B**, **I12** and **I12/B**.

All DFT calculations were carried out using the DFT/B3LYP method at the basis set 6-31G (d,p) for all **In/B** complexes. Since imaginary frequencies are absent, this could provide evidence for the geometrical stability of all H-bonded complexes. Figure 7 shows the geometrical structure of the bonded complexes of the base **B** with the azo acid **In** of n = 12 carbon atoms in the alkoxy chain.

As shown from Figure 7, although both base **B**, as well as the acid **I12**, are completely linear, the geometry of the formed complex is not completely linear. The formed complex has two parts. The first is linear, derived from the azopyridene moiety of the dinitrogen compound, and the other non-linear part is that of the nicotinate ester. The length of the complex derived from azo acid **I12** is 77.20 Å and its height is 15.01 Å to form a molecule with area 1158.57 Å^2^. The length of the linear part is 18.89 Å and that of the non-linear one is 54.28 Å. On the other hand, the angle of the non-linear part is 144°. The unsymmetrical geometry of the formed complex could be an explanation of the nematic mesophase formation. This geometry cannot permit the lateral interaction of the molecule to enhance the smectic mesophase; however, it leaves the terminal aggregation of the alkoxy groups to be predominated to enhance the nematic mesophase. The presence of the aromatic part of these supramolecular complexes could explain the high thermal stability and mesophase range, Tc = 230.1 and *Δ*T_N_ = 60.9 °C, respectively. On the other hand, neither the mesophase range nor their stability were affected by the length of the observed mesophase, and this could be another evidence of the effect of the π–π stacking of the aromatic moiety on the azo acid that can increase the stability of the enhanced mesophase [64,65,66,67].

#### 3.3.2. Effect of the H-Bonding and π–π Stacking on the Mesophase Behavior

The investigation of the impact of the H-bonding interactions on the mesophase stability and mesophase range can be achieved by a comparison of the mesomorphic behavior of the H-bonded **I12/B** complex with the individual azo acid **I12.** It has been reported [60] that the azo acid **I12** has smectic C and narrow nematic range (only ≈ 6 °C), however, the formation of the H-bonded complex **I12/B** induced the nematic range to be 60.9 °C. The induction of the wide nematic range could be attributed to the higher degree of the terminal aggregation of the alkoxy chains of the H-bonded complex with respect to the free acid. Moreover, the presence of the three aromatic rings of the dinitrogen compound **B** can also increase the range by extra parallel interactions of the aromatic rings of the H-bonded complex. Furthermore, the linear and planar geometry of the free azo acid **I12** enhances the smectic C mesophase with a very narrow range of the nematic phase due to the high degree of lateral interaction which facilitates close packing of the molecules to grow the more ordered smectic mesophase. However, the less linear geometry of the formed supramolecular **I12/B** complex decreases the chance of the lateral interaction with higher terminal aggregation forces to enhance the nematic mesophase of more random textures. 

The effect of the number of the aromatic rings that impacts the degree of the backing of the molecules is also studied with the previously reported supramolecular H-bonded complex of the same dinitrogen base **B** with dodecyloxybenzoic acid [68]. Our present compounds have seven aromatic rings instead of five rings of the alkoxybenzoic acid complexes. The higher number of the aromatic rings helps the strong π–π stacking of the molecules to increase the mesophase stability of the seven aromatic rings H-bonded complexes than that of the five aromatic rings of alkoxybenzoic acid complexes. The dodecyloxybenzoic acid H-bonded interactions of **II12/B** showed nematic stability 140.7 °C with respect to 230.1 °C of the azo acid **I12.** This data could be explained in terms of the higher degree of π–π stacking of the **I12/B** H-bonded complexes due to the incorporation of the phenylazo group than that of the five-ring **II12/B** system.

#### 3.3.3. Thermal Parameters

Values of thermal parameters were calculated with the same method at the same set for the 2:1 supramolecular H-bonded complexes **I6/B, I8/B, I10/B** and **I12/B**. All evaluated data are summarized in Table 2. The calculated data of Table 2 illustrate that the increment of the chain length highly affects the estimated stability of the supramolecular complexes, whereas the chain length increases the predicted energy deceases. The increment of alkoxy chain length has a negative effect on the predicted total energy and positive effect on the nematic mesophase stability of prepared H-bonded **In/B** complexes. 

#### 3.3.4. Frontier Molecular Orbitals, Dipole Moment and Polarizability

**Figure 8:**
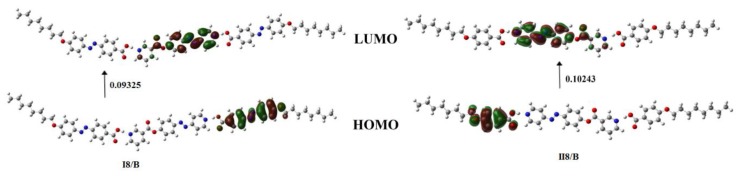
The estimated plots for frontier molecular orbitals of **I8/B** and **II8/B**.

**Figure 9:**
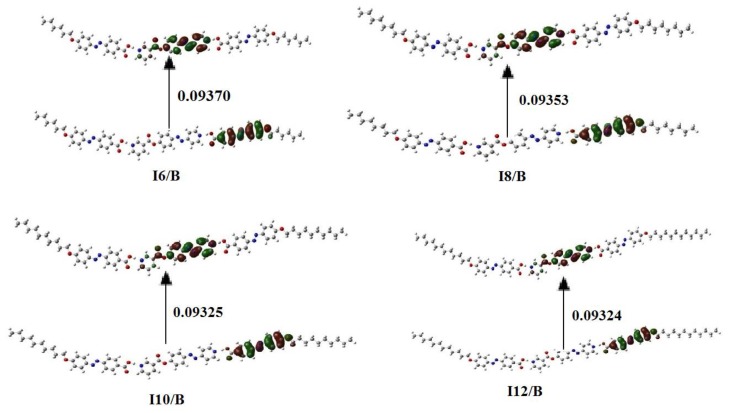
The estimated plots for frontier molecular orbitals of supramolecular **In/B** complexes.

Figure 8 and Figure 9 show that the estimated plots for frontier molecular orbitals HOMO (highest occupied) and LUMO (lowest unoccupied) of **In/B** and **II8/B**. From these figures, it is clear that the electron densities are mainly localized on the phenylazo acid (**In**) for HOMO while it shifted to the dinitrogen base in the case of LUMO. The energy difference between the frontier molecular orbitals (***Δ*E**) could be used in the prediction of the capability of electrons to transfer from HOMO to LUMO by any electron excitation process. The global softness (**S**) = **1/*Δ*E** is the parameter that predicts the polarizability as well as the sensitivity of the compounds for the photoelectric effects. The higher global softness of the compounds enhanced their photoelectric sensitivity as well as their polarizability. As shown from Table 3 and Figure 9, there is no significant effect of the length of the alkoxy chain on softness. However, the number of the aromatic ring has a high impact on the softness, and the supramolecular H-bonded complexes derived from the alkoxyphenylazobezoic **In**/**B** acids are softer than that of the alkoxybenzoic **IIn**/**B** acids. The extra conjugation of the seven aromatic ring supramolecular **In/B** complexes has higher polarizability than the five-ring complexes **IIn/B**. Moreover, the lower energy difference of **IIn/B** increases its polarizability to 949.74 of the complexes derived from octyloxyphenylazo acid (**I8/B**) instead of 661.95 for the octyloxybenzoic acid-based complex, **II8/B**. On the other hand, the length of the alkoxy chain of the acid component highly impacts the polarizability.

As we recently reported [63], the magnitude of the dipole moment affects the type of mesophase and its behavior. As shown in Table 3**,** the dipole moment of prepared complexes has an insignificant effect on the calculated dipole moment, and this could be illustrated in terms of the similar of the electronic nature of the alkoxy chains even for longer C-chains. However, the alkoxybenzoic acid **IIn/B** complexes are higher than that of the alkoxyphenylazobezoic acid **In/B** complexes. The higher dipole moment increases the lateral interaction with respect to the terminal one, and this could be a good explanation on the wide range nematic mesophase formation for the SMLC **In/B** complexes and with respect to their corresponding **IIn/B.**

#### 3.3.5. Molecular Electrostatic Potential (MEP)

**Figure 10:**
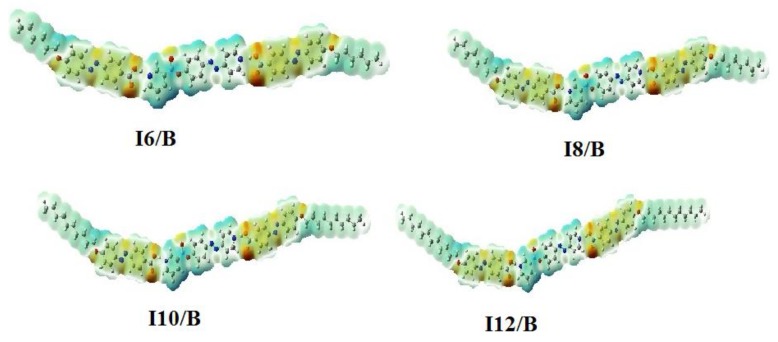
Molecular electrostatic potential (MEP) of **I6/B, I8/B, I10/B** and **I12/B**.

The charge distribution map for **In/B** was calculated with the same method on the same basis sets according to the molecular electrostatic potential (MEP; Figure 10). The negatively charged atomic sites (the red region) were estimated to be localized on carboxylate moiety of the alkoxyphenylazobezoic acid, while the moiety of the dinitrogen base as well as the alkyl chain was predicted to show the least negatively charged atomic sites (blue regions). As shown from Figure 10, the orientation of the charges is not affected by the length of the alkoxy chain, and consequently, this could be an illustration for the similarity of the mesophase behavior regardless of the chain length. The increment of the alkoxy chain length of **In/B** affects neither the orientation nor the amount of the charge distribution map, and this could illustrate the observed nematic phase for all H-bonded supramolecular complexes due to more end-to-end aggregation of the terminal alkoxy chains.

### 3.4. Photophysical Behavior

**Figure 11:**
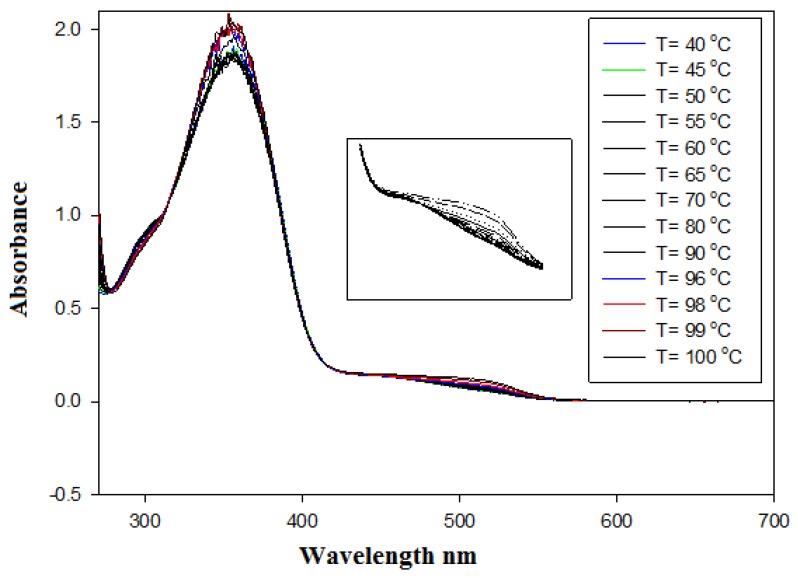
UV absorption spectrum of SMHB complex **I12/B** in DMF with a heating range from 25 to 100 °C.

The photophysical investigation of the **I12/B** complex as an example of a supramolecular H-bonded mixture was carried out by measuring UV–vis spectra. A solution of C = 1.8·10^−6^ mol/L in DMF was used to record spectrophotometric absorption spectra bands at a temperature range of 298–398 K. All photoisomerization absorption bands’ results are graphically represented in Figure 11. As shown in Figure 11, the SMHB **I12/B** complex has maximum absorption bands at ~361 nm and ~514 nm with a small shoulder at ~460 nm. The absorbance slightly increases with the temperature range. The highly delocalized electronic systems and π–π* transitions, due to the presence of conjugated dinitrogen linking parts with high molar absorption coefficient (ε = 10.4·10^5^ L mol^−1^cm^−1^), were recognized to the maximum absorption in the present H-bonding interactions. Moreover, the absorption and the intensity of the peak in the absorption spectrum are depending on the molecule which absorbs the light of a given wavelength. The absorption spectra of the complex showed maxima bands at 361 nm which attributed to the electronic transition from the highest occupied molecular orbitals (HOMO) to lowest unoccupied molecular orbital (LUMO) π–π transition [69,70,71].

## 4. Conclusions

Herein, we have reported the synthesis of new supramolecular H-bonded complexes of seven aromatic rings dipyridine-based derivative. Mesomorphic and optical characterizations were carried out by DSC, POM and UV spectroscopy. Interestingly, the orientations of dinitrogen atoms in the base moiety play an important role in the observation of enantiotropic nematic mesophase with broad nematogenic stability range for all mixtures. All these findings illustrate the purposes of the selection of azo linkers and explain how they could possess excellent characteristics to be of great potential and as valuable candidates for developing new applicable nematogenic architectures. Theoretical calculations provided information to understand the experimental results in terms of the relationships of the thermal and geometrical parameters. It was found that the π–π stacking of the aromatic moiety in the phenylazo acid plays an effective role in the wide mesomorphic thermal stability. Moreover, the DFT results explained how the aspect ratio of the complexes under investigation enhanced the mesophase ranges compared with our previously reported H-bonded complexes of the same dinitrogen base with short alkoxy chain arms. Further, the photophysical studies revealed for all complexes involves isomerization.

## Figures and Tables

**Table 1:** Phase transition temperatures (°C), enthalpy of transitions (kJ/mol) and normalized transition entropy of supramolecular **In/B** complexes.

System	*T*_Cr-N_	*ΔH*_Cr-N_	*T*_N-I_	*ΔH*_N-I_	*ΔS/R*
**I6/B**	168.5	47.65	241.2	4.8	1.12
**I8/B**	153.1	57.38	237.4	5.43	1.28
**I10/B**	163.8	61.49	234.0	6.08	1.44
**I12/B**	169.2	64.08	230.1	8.57	2.05

Abbreviations: ***T*_N-I_**: Nematic to isotropic liquid transition. ***ΔH*****_N-I_**: Nematic to isotropic liquid phase transition. ***ΔS/R***: Normalized entropy transition.

**Table 2:** Thermal Parameters (Hartree/Particle) of I6/B, I8/B, I10/B, I12/B and II8/B.

Parameter	I6/B	I8/B	I10/B	I12/B	II8/B
**E_corr_**	1.025964	1.140069	1.254160	1.368322	0.959290
**ZPVE**	−3168.161489	−3325.295728	−3482.429991	−3639.564172	−2644.501692
**E*_tot_***	−3168.093627	−3325.222380	−3482.351174	−3639.479910	−2644.441531
**H**	−3168.092683	−3325.221436	−3482.350230	−3639.478966	−2644.440586
**G**	−3168.288686	−3325.431743	−3482.573992	−3639.716414	−2644.616210

Abbreviations: E_corr_: Thermal correction to energy; ZPVE: Sum of electronic and zero-point energies; E_tot_: Sum of electronic and thermal energies; H: Sum of electronic and thermal enthalpies; G: Sum of electronic and thermal free energies.

**Table 3:** FMO Energies a.u., Polarizability, α, and Dipole Moment μ (Debye) of I6/B, I8/B, I10/B, I12/B and II8/B.

Parameter	I6/B	I8/B	I10/B	I12/B	II8/B
**E_LUMO_**	−0.12678	−0.12686	−0.12703	−0.12704	−0.12450
**E_HOMO_**	−0.22048	−0.22039	−0.22028	−0.22028	−0.22693
***Δ*E_HOMO-LUMO_**	0.0937	0.09353	0.09325	0.09324	0.10243
**1/*Δ*E**	10.67236	10.69176	10.72386	10.72501	9.762765
**μ Total**	1.7921	1.7962	1.7948	1.7348	2.6037
**Polarizability α**	901.07	949.74	997.65	1045.29	661.95

## Supplementary Materials

The following are available online. Scheme S1: Preparation of 4-(2-(pyridin-4-yl)diazenyl)phenyl nicotinate (I), Table S1: Phase transition temperatures (°C), enthalpy of transitions (kJ/mol), and transition entropy of supramolecular complex IIn/B.
