# Supplementary material for: Induced Wide Nematic Phase by Seven-Ring Supramolecular H-Bonded Systems: Experimental and Computational Evaluation
Source: Molecules. 2020 Apr 7;25(7):1694. doi: 10.3390/molecules25071694 (PMC7180779; doi:10.3390/molecules25071694)
Supplement: Supplementary file 1 [file molecules-25-01694-s001.pdf]

Supplementary Data

Induced wide nematic phase by seven rings supramolecular H-bonded systems; Experimental and computational evaluation

Latifah Abdullah Alshabanah [1], Laila A. Al-Mutabagani [1], Hoda A. Ahmed [2,3,*], Mohamed Hagar [3,4,*]

[1] College of Science, Chemistry Department, Princess Nourah bint Abdulrahman University, Riyadh, 11671, Saudi Arabia. (L.A.A.M) Laalmutbagani@pnu.edu.sa, (L.A.A.) Laalsabanah@pnu.eud.sa

[2] Faculty of Science, Department of Chemistry, Cairo University, Cairo, 12613, Egypt. (H.A.) ahoda@sci.cu.edu.eg.

[3] Chemistry Department, College of Sciences, Yanbu, Taibah University, Yanbu 30799 Saudi Arabia

[4] Faculty of Science, Chemistry Department, Alexandria University, Alexandria, 21321, Egypt. (M.H.) mohamedhaggar@gmail.com

* Correspondence: mhagar@taibahu.edu.sa (M.H.); ahoda@sci.cu.edu.eg (H.A.)

Material and methods

Nicotinic acid, *N,N′*-dicyclohexylcarbodiimide (DCC), phenol, 4-aminopyridine, 4-dimethylaminopyridine (DMAP) and 3-aminopyridine were purchased from Aldrich (Wisconsin, USA). All solvents used are pure grade and purchased from Aldrich (Wisconsin, USA).

TA Instruments Co. Q20 Differential Scanning Calorimeter (DSC; USA) were using for calorimetric measurements. The DSC was calibrated using the melting temperature and enthalpy of indium and lead. DSC investigation was carried out for small samples (2–3 mg) placed in aluminum pans. All measurements were achieved at a heating rate of 10°C/min in inert atmosphere of nitrogen gas (30 ml/min) and all transition recorded from the second heating scan.

Transition temperatures for the individual components and their 2:1 associated complexes, were determined by DSC, and the types of the mesophase identified by a standard polarized optical microscope (POM, Wild, Germany) attached with Mettler FP82HT hot stage. The temperature is measured by thermocouple attached to the temperature controller. Measurements were made twice and the results have accuracy in transition temperature within ± 0.2°C.

Characterizations

Purity of **I** was checked with thin-layer chromatography using TLC and elemental analyses. The structure was confirmed by FTIR (Nicolet iS 10 Thermo scientific), and ^{1}H-NMR spectroscopy (Varian EM 350L 300 MHz spectrometer, Oxford, UK).

Computational Method and calculations

The theoretical calculations for the investigated compounds were carried out by Gaussian 09 software [1]. DFT/B3LYP methods using 6-31G (d,p) basis set was selected for the calculations. The geometries were optimized by minimizing the energies with respect to all geometrical parameters without imposing any molecular symmetry constraints. The structures of the optimized geometries had been drawn with Gauss View [2]. Moreover, the calculated frequencies were carried out using the same level of theory. The frequency calculations showed that all structures were stationary points in the geometry optimization method with none imaginary frequency.

Preparation of dipyridine-based derivative

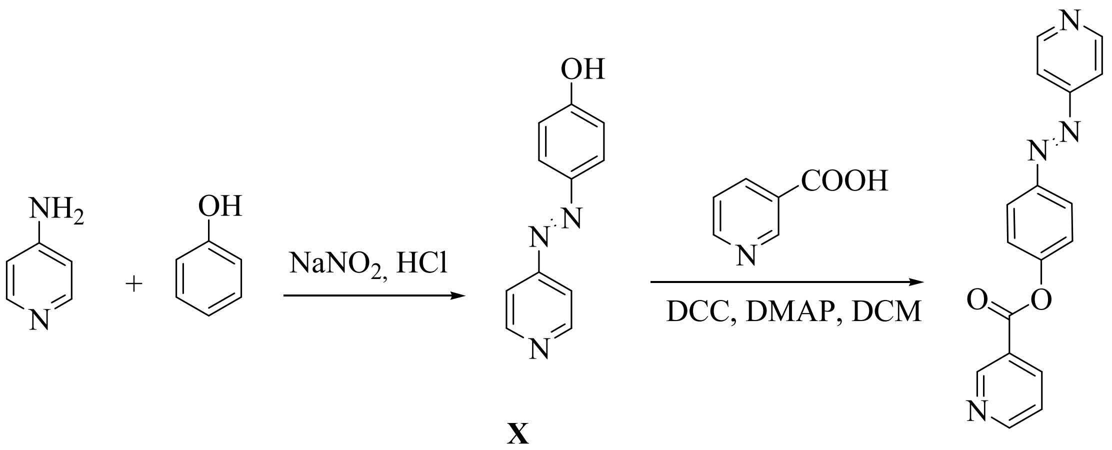

Scheme S1. Preparation of 4-(2-(pyridin-4-yl)diazenyl)phenyl nicotinate (**I**).

Synthesis of 4-(2-(pyridin- 4-yl)diazenyl)phenol (**X**)

A mixture of (2.5 g, 26.5 mmol) phenol in sodium hydroxide 10% (50 ml) was allowed to cool to 0 ^{o}C. Another solution of 4-aminopyridine (2.4 g, 27 mmol) in 6 molar HCl was cold. The solution of was added drop wise to a cold aqueous solution of sodium nitrite (2.0 g, 30 mmol) in H_2O (20 ml). To the former solution, we added the diazonium solution prepared in the later step with stirring within 10 minutes and keeping the reaction temperature below 5 ^{o}C. Subsequently, the complete precipitation of the product was performed by neutralization of the reaction mixture with sodium carbonate till pH ≈ 6. The obtained orange dye was purified by recrystallization from hot aqueous ethanol (1:1).

Synthesis of 4-(2-(pyridin- 4-yl)diazenyl)phenyl nicotinate (**I**)

An equal molar ratios of 4-(2-(pyridin-4-yl)diazenyl)phenol (**1**) (1.99, 0.01 mole) and nicotinic acid (1.23 g, 0.01 mole) was dissolved in 25 ml dry methylene chloride. A catalytic amount of 4–dimethylaminopyridine (DMAP) and 0.02 mole of *N*, *N′*–dicyclohexylcarbodiimide (DCC,) was added. The reaction mixture was kept stirring for 72 hours at room temperature. The separated byproduct, *N,N*-dicyclohexylurea, was filtered off. The evaporation of the filtrate afforded the ester derivative, The product was recrystallized twice from ethanol (**Scheme 1**).

4-(2-(pyridin- 4-yl)diazenyl)phenyl nicotinate (**I**)

Yield: 92.7 %; mp 148.1 °C, FTIR (ύ, cm^{-1}): 3049 (=CH stretching), 1726 (C=O), 1577 (C=N), 1489 ($C–O_{Asym}$), 1275 ($C\text{-}O_{Sym}$). ^{1}H NMR (400 MHz, $CDCl_3$): δ/ppm: ^{1}H NMR (400 MHz, $CDCl_3$): δ/ppm: 9.45 (s, 1H, Py), 8.92 (d, *J* = 4.7 Hz, 1H, Py), 8.87 (d, *J* = 5.0 Hz, 2H, Py), 8.51 (d, *J* = 7.9 Hz, 1H, Py), 8.13 (d, *J* = 8.6 Hz, 2H, Ph), 7.87 (d, *J* = 5.3 Hz, 2H, Py), 7.53 (dd, *J* = 8.0, 4.9 Hz, 1H, Py), 7.49 (d, *J* = 8.6 Hz, 2H, Ph). ^{13}C NMR (101 MHz, $CDCl_3$) δ/ppm: 163, 158, 154, 154, 151, 150, 149, 138, 125, 124, 123, 117. Elemental analyses: Found (Calc.): C, 67.09 (67.10); H, 3.94 (3.97); N, 18.38 (18.41).

Previously reported Dinitrogen alkoxy SMHBC

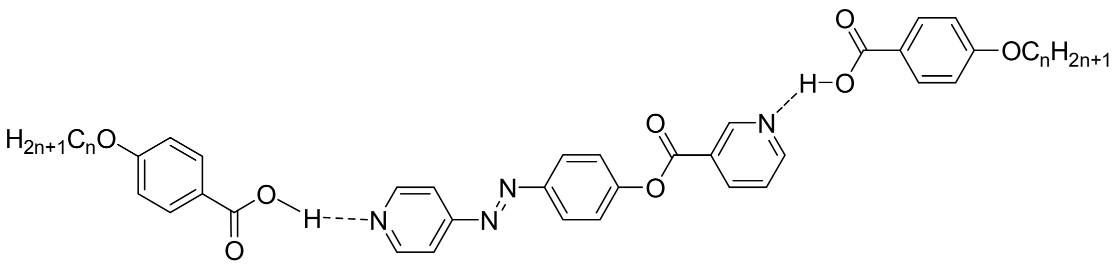

IIn/B

Table S1.Phase transition temperatures (°C), enthalpy of transitions (kJ/mol), and transition entropy of supramolecular complex **IIn/B**

System	T_{Cr-N}	ΔH_{Cr-N}	T_{N-I}	ΔH_{N-I}	$\Delta S/R$
**II6/B**	102.9	44.68	143.3	3.74	3.14
**II8/B**	94.7	50.80	144.0	5.35	4.47
**II10/B**	94.9	41.48	143.7	3.27	2.74
**II12/B**	92.8	49.05	140.7	3.91	3.34
**II16/B**	95.8	53.94	139.2	5.76	4.98

Abbreviations: T_{Cr-N}= Crystal to nematic phase transition; T_{N-I} = Nematic to isotropic liquid transition. ΔH_{Cr-N}= Crystal to nematic phase transition; ΔH_{N-I}= Nematic to isotropic liquid phase transition. $\Delta S/R$ = Normalized entropy of nematic transition.

References

1. Frisch, M.; Trucks, G.; Schlegel, H. B.; Scuseria, G.; Robb, M.; Cheeseman, J.; Scalmani, G.; Barone, V.; Mennucci, B.; Petersson, G., Gaussian 09, revision a. 02, gaussian. *Inc., Wallingford, CT* **2009,** 200.
2. Dennington, R.; Keith, T.; Millam, J., GaussView, version 5. **2009**.
